# Challenges to ART market: a Polish case

**DOI:** 10.1007/s11019-014-9573-x

**Published:** 2014-06-03

**Authors:** Anna Alichniewicz, Monika Michałowska

**Affiliations:** 1 Uniwersytet Medyczny w Łodzi, Łódź, Poland; 2 al. Lindley 6, 90-131 Łódź, Poland; 3 ul. Lindley 6, 90-131 Łódź, Poland

**Keywords:** ART market, Poland, Commercialization, Altruism

## Abstract

In the paper we are analyzing the Polish ART market. It can be noticed that the lack of legal regulation has resulted in many discrepancies among the policies adopted by various ART agencies. The social acceptance of ART procedures available mostly in private clinics led to growing commercialization of the Polish ART market. Additionally, the language of gift and altruistic rhetoric that are overwhelmingly employed by ART agencies reveals hypocrisy of the Polish ART market.

The Polish ART market is relatively young. The first in vitro fertilization (IVF) successfully performed in Poland was reported in 1987 (Szamatowicz et al. 1988), thus it seems interesting to take a closer look at its structure. In our paper, we are going to shed light on some idiosyncrasies of the Polish ART market as well as the challenges it is facing. Our goal is to provide a description of the Polish ART realm as well as to analyze the phenomena we find especially characteristic.

The first phenomenon we would like to point out is a rising number of agencies, both domestic and foreign that has been offering ART procedures to Polish patients in the last years (Table 1). In the years 2008–2011 a constant increase in the number of IVF procedures was observed (Table 1). Also, the number of additional procedures, that is embryo donation (Table 2) and egg donation (ED, Table 3) rose by 100 %. Moreover, since 2008 we have witnessed an increase in the number of clinics reporting to Fertility and Infertility Section (SPIN) of the Polish Gynecological Society (PTG, Table 1) which is the European Society of Human Reproduction and Embryology (ESHRE) data provider, even though the reporting still remains voluntary. Being aware of the fact that reporting could contribute to their credibility, some clinics announce it on their websites: “Gameta is a centre reporting the results of the effectiveness of assisted reproduction techniques since the beginning of the European Society of Human Reproduction and Embryology (ESHRE) register” (Gametaa 2014).

**Table 1:** The number of ART clinics in Poland 2008–2011

Year of the survey	Total number of ART clinics	Number of ART clinics reporting to the National Register	Number of the clinics: <100 cycles	100–199 cycles	200–499 cycles	500–999 cycles	≥1.000 cycles
2008	Unknown	25	4	6	6	4	2
2009	31	25	8	2	9	5	1
2010	38	29	10	2	10	6	1
2011	38	30	6	6	11	6	1

The reports of SPIN [The surveys are carried out for *the European IVF-Monitoring (EIM) Consortium for the European Society on Human Reproduction and Embryology (ESHRE)*. The data is collected and analyzed by SPIN (Fertility and Infertility Section) of PTG (the Polish Gynecological Society)] (Fertility and Infertility Section) of PTG (the Polish Gynecological Society) 2008–2011 http://spin.org.pl/eim-europejski-monitoring-wynikow-ivf/. The reports are published with a 2-year delay

**Table 2:** The number of ART procedures in Poland 2008–2011 (IVF cycles/aspirations/pregnancies/deliveries; embryo donation)

Year	Initiated cycles IVF	Aspirations (IVF)	Pregnancies* (IVF)	Deliveries* (IVF)	Embryo donation
2008	282	267	96	Unknown	No data
2009	285	273	96	Unknown	123
2010	347	335	125	56	241
2011	501	481	176	109	251

The reports of SPIN (Fertility and Infertility Section) of PTG (the Polish Gynecological Society) 2008–2011 http://spin.org.pl/eim-europejski-monitoring-wynikow-ivf/. The reports are published with a 2-year delay

Pregnancies*/Deliveries* SPIN uses “the WHO/ICMART definition of clinical pregnancy: evidence of pregnancy by clinical or ultrasound parameters (ultrasound visualization of a gestational sac). It includes ectopic pregnancy. Multiple gestational sacs in one patient are counted as one clinical pregnancy. Deliveries include those resulting in a live birth and/or stillbirth”. http://humrep.oxfordjournals.org/content/24/11/2683.full.pdf+html

**Table 3:** Women’s age and ED (egg donation)

Year	Woman’s age	
	≤34	35–39
2008	66	96
2009	70	120
2010	82	114
2011	160	189

The number of egg recipients 2008–2011

The reports of SPIN (Fertility and Infertility Section) of PTG (the Polish Gynecological Society) 2008–2011 http://spin.org.pl/eim-europejski-monitoring-wynikow-ivf/

The reports are published with a 2-year delay

In ED the age refers to the recipient

As far as the question of social acceptance of this type of medical service is concerned, we would like to refer to the attitudes presented by two cohorts. As a part of research we are currently doing on various aspects of the ART realm in Poland, in 2013 we carried out a study of views on IVF, ED and preimplantation genetic diagnosis (PGD), in which we asked 178 undergraduate and PhD students of one of Polish medical universities about their attitude to the aforementioned procedures. The survey revealed that 90 % of the respondents are of the opinion that IVF should be legal, 82 % of the respondents are of the opinion that PGD (screening-out) should be legal, 79 % of the respondents are of the opinion that ED should be legal. The other group whose opinion on ART was investigated was a group of patients of one of Polish ART clinics. The results of the study were published in 2012 and showed a high acceptance of these kinds of infertility treatment. For example, out of 213 female patients who took part in the study 76.6 % were of the opinion that ED should be allowed (Dembińska 2012).

At the beginning of the development of ART in the 1980s there were some controversies over whether this type of medical practice, so deeply embedded in entirely private matters, should be legally regulated. Nevertheless, next decades witnessed a rapid progress in ART accompanied by two social phenomena: a rising social approval and some signs of commercialization of ART service (Braun and Schultz 2012). It could be argued that these phenomena are responsible for a growing recognition of the indispensability of some kind of legal regulation (Fournier et al. 2013). Nowadays, a similar tendency can be observed in Poland, where a general awareness of the necessity of putting ART procedures into some legal framework emerged when this medical practice gained more interest (Kulawik 2012). However, despite the fact that this need has been discussed for several years, there are still no laws regulating ART procedures in Poland.

It should be noted that despite the inability to establish any feasible legal framework, a call for ethical guidelines has paradoxically appeared more fruitful and has already resulted in several proposals. One of the proposals which was issued by the Polish Chamber of Physicians and Dentists (PCPD) in September 2009 (PCPDa 2014) and reconfirmed in January 2013 (PCPDb 2014) presented a general stance of PCPD with regard to a cluster of ethical problems of contemporary medicine, including ART procedures. The recognition of ART as one of the most important problems of today medicine that should be ethically and legally addressed as well as a clear call for establishing a legal framework for ART procedures and a legal protection of infertile couples make their proposal an important voice in the Polish debate. It can be noticed, however, that the guidelines concerning ART covering only some selected aspects of the procedures are laconic and no ethical rationale is given behind them. Worth mentioning is also the fact that it is “the integrity and dignity of human embryos” that is emphasized in PCPD’s statement. As far as IVF is concerned, the prohibition of spare embryos creation, as well as the prohibition of all kinds of PGD, that is, both screening-in, including screening for human leucocyte antigen (HLA), and screening-out are recommended. Furthermore, in PCPD’s view IVF should not be available for postmenopausal patients. The aforementioned guidelines seem rather conservative, especially in comparison to a more liberal stance presented in two statements issued by the Polish Bioethical Committee (PBC) (PBC 2014). PBC’s proposals allow cryopreservation of gametes and embryos, PGD that covers most forms of screening-out, although excludes the ones for late-onset diseases as well as for HLA. According to PBC, state funding of IVF and PGD should be guaranteed. In their view also extra partnership donation of gametes and embryos (that is donation of gametes or embryos by one couple to another) should be considered ethically justified. However, some essential issues, like standards of obtaining informed consent, as well as guidelines on collection and reporting of data are not covered by the document. It also lacks ethical guidelines on egg donation, especially with regard to the question of anonymity of gamete donors and the standards of the procurement and later use of gametes that are ART vital procedures. Comparing the two documents issued by PBC, it could be noticed that whereas the one concerning PGD is quite elaborate, the one concerning IVF is far from being comprehensive.

Although the guidelines issued by PCPD and PCB have been widely discussed in the physicians and bioethicists’ milieus, and as such they could be expected to contribute to setting ethical standards of ART, we observe that so far they have had little impact on this practice in Poland. It seems that for the time being ART service in Poland is regulated mostly, if not entirely, by the market, so there are still some inconsistencies regarding specific policies adopted by ART agencies.

It is especially visible in the policies concerning gamete donation and the instructions for prospective female donors that are available on the websites of ART agencies. For instance, some ART agencies hold that only an IVF patient can become an egg donor and that it does not decrease her chances of pregnancy (Invictaa 2014). In other words, according to these agencies ED can only be combined with the IVF treatment. There are, however, other agencies that insist that the IVF patient should definitely not become an egg donor, since it would have a detrimental impact on her pregnancy chances (Gametab 2014). The instruction for potential egg donors is often associated with some additional information concerning various forms of compensation provided for egg donors. Generally, agencies operating on the Polish ART market offer three forms of financial recompense, that is egg sharing, compensation and reimbursement of expenses, which are the forms approved also in other European countries (Gürtin and Vayena 2012).

It is worth investigating what is actually offered under these headings by the agencies active on the Polish ART market. The so-called ‘egg sharing’ means a scheme where egg donors are offered an IVF procedure at a reduced price (Invictab 2014). The scheme is advertised as a special offer by which IVF is made affordable for the less-affluent. Compensation is offered as a form of financial recompense awarded to egg donors for the inconveniences associated with their visits to ART clinic (Gyncentruma 2014). Another form offered to egg donors is reimbursement for their travel and accommodation costs (Gametac 2014). Generally, two basic schemes can be found: egg donors are compensated for their time, lost earnings and discomfort, or reimbursed for the cost of travel, medication and maintenance during the procedure. It seems that the former scheme relates to the ‘subjective’, whereas the latter to the ‘objective’ sides of the burden imposed on egg donors. Although compensation and reimbursement are presented as two different schemes, it would be very difficult to tell the difference between them on the basis of the information provided by ART clinics on their websites. It can be argued that the main difference lies in words, that is, the agencies just use different labels to name the same thing, namely payment for the expenditures and inconveniences.

What we find especially interesting to investigate is a call for altruistic donation encountered on the ART agencies’ websites (Invictac 2014). The reference to altruistic gift and help appears not only under the heading of ‘altruistic egg donation’, but also in the schemes where financial recompense is openly offered. You could expect a scheme presented as altruistic egg donation to differ essentially from the aforementioned schemes where financial recompense is involved. But it is not the case. Although the altruistic deed is generally meant as selflessly motivated help accompanied by dedication and even a sort of self-sacrifice, the so-called altruistic oocyte donation is also to be reimbursed. Therefore, it can be noticed that with regard to ED the language of gift is often employed by ART agencies. Analysis of the notion of altruism functioning in the ART realm raises also a more fundamental question.

Empirical data show that IVF procedures involving ovarian stimulation and oocyte retrieval may have detrimental outcomes for women’s health, including the most serious ones, like ovarian hyperstimulation syndrome (OHSS) that can even have life-threating consequences (Haimes et al. 2013; McLeod and Baylis 2007). Given the data, we can wonder whether asking a patient to donate her oocytes is fair. On the ART agencies’ websites becoming an egg donor is presented as a manifestation of empathy, solidarity and benevolence, that is, a manifestation of virtues traditionally ascribed to women. The question is arising whether this set of virtues reflects genuine attitudes prevailing among women or maybe it is rather a part of traditional Western views on gender roles. Erik Malmqvist and Kristin Zeiler argue that the highly demanding ideal of feminine altruism may be a result of the process of socialization in which gender “cultural norms are habitually incorporated” (Malmqvist and Zeiler 2010, p. 144). The process of ‘imprinting’ may put women having to decide whether to donate their gametes or not in a difficult position, because these norms may become so deeply incorporated and self-concealed that they turn out to be totally determining. If this is the case, the decision made by a potential oocyte donor may not reflect her genuine wishes. It can also be argued that by referring to cultural gender stereotypes, the ‘ideology’ of ART agencies supports a highly demanding model of feminine moral ideal, which in this case serves perfectly the interests of ART agencies. Given that the language of gift and the call for ‘solidarity of ovaries’ appear also in the information about egg sharing, it could be suggested that it is a sort of manipulation. The so-called egg sharing might become a camouflage for a new form of reproductive exploitation, since in that way women, who otherwise could not afford an IVF procedure, are pressed to donate their oocytes, whereas the whole procedure is disguised as altruistic egg donation. The ‘imprinted altruism’ we are referring to as well as potential exploitation faced by female patients in the ART realm have been the subject of current debate (Charles 2010; Haimes et al. 2012; Scully et al. 2012; Solinger 1998; Waldby 2008). We have also argued elsewhere extensively that such a call for altruism in the domain of ART may be highly persuasive and can put female patients at risk of exploitation (Alichniewicz and Michałowska 2014). Moreover, it has been claimed that a presupposition of female altruism stemming from gender stereotypes deeply rooted in Western culture resulted in making women invisible in the reproduction debate (Alichniewicz and Michałowska 2014; Kulawik 2012). “The lady vanishes” as Donna Dickenson eloquently put it (Dickenson 2007).

Investigating the ART market in Poland we observed that there is also a discrepancy between the data concerning success rate provided by ART agencies and SPIN. For instance, Gyncentrum inform that their ICSI success rate for 2010 was 42 % (but does not specify whether the percentage refers to the number of pregnancies or deliveries), whereas according to SPIN data for the same year, the average ICSI success rates were 34 and 26 % for pregnancies and deliveries, respectively (Gyncentrumb 2014; Table 4).^1^ Our attention was also drawn to the statistics regarding IVF/ICSI success rate for 2009 provided by INVICTA. They inform that the respective rates were 47 % for INVICTA, 41,6 % for Poland and 33 % for Europe, whereas according to ESHRE in 2009 “On average, pregnancy rates were 28.9 % (+0.4 % compared with 2008) and 28.7 % (−0.2 %) per aspiration for IVF and ICSI, and 20.9 % per thawing for frozen embryo replacement (FER) (+1.6 %)” (Ferraretti et al. 2013; Invictad 2014). Moreover, one of the clinics, Gameta openly admit that statistics published by the clinics can be misleading as they may not reflect real outcomes of the procedures, so—as a matter of fact—they are useless. Notwithstanding this confession, they declare that due to a wide spectrum of procedures available for their patients, the overall rate of pregnancies in Gameta is 90 % (Gametad 2014).

**Table 4:** The number of ICSI, pregnancies and deliveries in Poland 2008–2011

Year	Initiated cycles ICSI	Pregnancies*	Deliveries*
2008	6,462	2,453	Unknown
2009	7,566	2,757	Unknown
2010	8,621	2,937	2,233
2011	9,510	3,244	2,257

The reports of SPIN (Fertility and Infertility Section) of PTG (the Polish Gynecological Society) 2008–2011 http://spin.org.pl/eim-europejski-monitoring-wynikow-ivf/ The reports are published with a 2-year delay

Pregnancies*/Deliveries* SPIN uses “the WHO/ICMART definition of clinical pregnancy: evidence of pregnancy by clinical or ultrasound parameters (ultrasound visualization of a gestational sac). It includes ectopic pregnancy. Multiple gestational sacs in one patient are counted as one clinical pregnancy. Deliveries include those resulting in a live birth and/or stillbirth”

The majority of the agencies operating on the Polish ART market provide also an English version of their websites. It seems obvious that you would expect to find exactly the same set of offers, information and instructions for patients in the both versions. But this is not the case. The comparison of the Polish and English versions reveals substantive differences. Although, at the first sight the two versions seem to provide the same information concerning ED program, a more insightful reader will notice that some important information is missing in the English one. For instance, the English version does not mention any compensation an egg donor is entitled to (Gyncentrumc 2014). Moreover, the English version provides no information concerning the ED program that is extensively described in the Polish version of the website. The sets of FAQs contained in the Polish and English versions are also quite different (Gametae 2014).

It can be held that the fact that in Poland ART procedures are available only in private practice and are not reimbursed by the National Health Fund^2^ have resulted in their gradual commercialization. As a very symptomatic phenomenon can be regarded the way one of Polish fertility clinics advertises their service, namely as ‘In Vitro All Inclusive’ (Invictae 2014). The ‘all inclusive’ offer not only covers all possible ART procedures available at the clinic, but also—as an extra ‘bonus’—some discount is provided for the ‘clients’ who have decided on that option. Thus, it seems that ART procedures in Poland have been becoming just another type of a commercial service that tries to meet the needs and demands of modern society.

To sum up, it can be argued that the Polish ART market is characterized by rising commercialization of the service disguised by the language of gift. The fact that the commercialization is accompanied by altruistic rhetoric discloses hypocrisy the Polish ART business is affected by.

## Footnotes

1 It seems also worth mentioning that Gyncentrum did not reply to our inquiries about the statistics provided on their website.

2 Although the debate on whether ART procedures should be reimbursed by the National Health Fund has already begun several years ago, it still remains inconclusive. It is probably the awareness of the expectations of the Polish society that has recently triggered the Polish government to a rather surprising turn: despite the lack of legal framework, the IVF program covering the procedure for 15,000 couples by the National Health Fund was announced for years 2013 – 2016.
